# Supplementary material for: Monoclonal Antibody-Based Immunosensor for the Electrochemical Detection of Chlortoluron Herbicide in Groundwaters
Source: Biosensors (Basel). 2021 Dec 13;11(12):513. doi: 10.3390/bios11120513 (PMC8699797; doi:10.3390/bios11120513)
Supplement: Supplementary file 1 [file biosensors-11-00513-s001.zip › biosensors-1478526-supplementary.pdf]

Supplementary information

1. Electrochemical Characterization of TMB on SPCE

Tetramethylbenzidine (TMB) and H_2O_2 are the co-substrates of HRP, which is used as secondary antibody label. The electrochemical behaviour of TMB on SPCE was studied by cyclic voltammetry (CV) to determine the was first used to investigate the electrochemical characteristics of TMB on a SPCE, by scanning the potential between +1.0 and −1.0 V vs Ag/AgCl at a scan rate of 50 mV.s^{-1} (Figure S1).

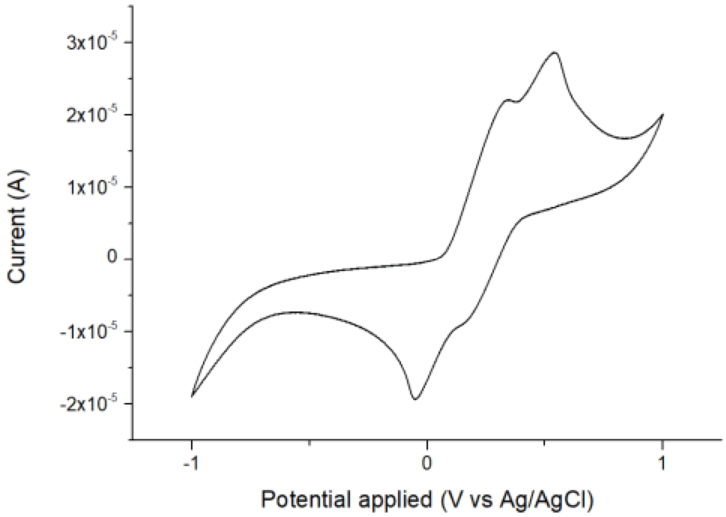

Figure S1. Cyclic voltammogram showing TMB oxidation and reduction waves on bare SPCE.(scan from +1.0 to −1.0 V vs Ag/AgCl at a scan rate of 50 mV.s^{-1}).

As previously described in literature, TMB undergoes a two-electron oxidation-reduction process [34]. In our system, it is characterized by two oxidation peaks at 303 mV and 540 mV vs Ag/AgCl, and two reduction peaks at 198 mV and -50 mV vs Ag/AgCl. Based on these observations and according to previous works [35], an applied potential of −200 mV vs Ag/AgCl was chosen for subsequent chronoamperometric experiments, with the aim of reducing efficiently the oxidized TMB formed on the electrode surface.

2. Optimization of Streptavidin Coating

Measurements were first performed for optimizing the concentrations of streptavidin used for working electrode modification. Different concentrations of streptavidin were tested as well as increasing concentrations of biotinylated chlortoluron, while concentrations of monoclonal and secondary antibody were set at 3 and 10 µg.mL^{-1}, respectively.

As shown in Figure S2, a streptavidin concentration of 50 µg.mL^{-1} led to non proportional responses to increasing concentrations of biotinylated chlortoluron. Using a concentration of 25 µg.mL^{-1} led to coherent results but high standard deviations were obtained between electrodes. On the contrary a minimal streptavidin concentration of 5 µg.mL^{-1} was sufficient to obtain a ratio between positive and negative controls (M_{t+} / M_t) higher than 5, with low standard deviations. Based on the fact that low

concentrations of biotinylated chlortoluron are mandatory for developing a sensitive immunosensor, concentrations of streptavidin and conjugated chlortoluron of 5 µg.mL^{-1} were selected for further assays.

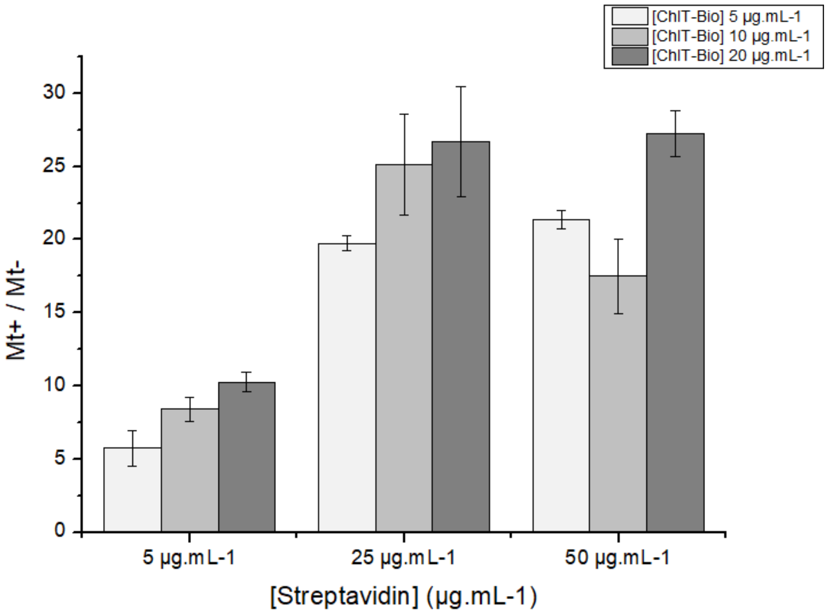

Figure S2. Ratio between positive control (Mt+) and negative control (Mt−) signals as a function of streptavidin concentration (5, 25 and 50 µg.mL^{-1}) and biotinylated chlortoluron concentration (ChlT-Bio ; 5, 10 and 20 µg.mL^{-1}).
